# Insights with consensus on Abductor Pollicis Longus from the Central Indian population at Nagpur, Maharashtra

**DOI:** 10.6026/973206300191111

**Published:** 2023-11-30

**Authors:** Vishwajit Deshmukh, Shweta Talhar, Gayatri Muthiyan, Payal Kasat, Kirubhanand Chandrasekaran, Bharat Sontakke

**Affiliations:** 1 Department of Anatomy, AIIMS, Nagpur, Maharashtra, India; 2 Department of Anatomy, MGIMS, Sewagram, Maharashtra, India; 3 Department of Anatomy, Dr B C Roy Multispecialty Medical Research Center, Kharagpur, West Bengal, India

**Keywords:** Abductor pollicis longus, anatomical, variations, anatomical snuff box

## Abstract

The anatomical snuff box is bounded laterally by the Abductor pollicis longus (APL) and the Extensor pollicis brevis (EPB) tendons. The variation in insertion of extensor tendons at the forearm and wrist is an interesting phenomenon. Therefore, a sound knowledge of such variations in extensor tendons is essential to know the consequence of tendon injury during implementation of its repair. Hence, we studied 48 formaldehyde fixed forearms of Indian-origin cadavers with age groups ranging from 33 to 67 years from the Central Indian population at Nagpur, Maharashtra, India. Variation in the insertion of APL was checked by tracing the tendon till its insertion. APL muscle was found with single tendon in 20 forearms, double in 9, triple in 8 and quadruple in 5 and five (maximum) in 6 forearms. In 93% (n=45), the APL tendon was inserted into the first metacarpal bone and in 7% (n=03), it was inserted into the trapezium bone. No variation was noted in the EPB tendon. Data shows that there are accessory slips in the APL tendon, differing from the standard description. Thus, data provides awareness of such potential variation among operating surgeons for better management of the diseased during dissection.

## Background:

The anatomical snuff box or tabatiere anatomique is a French term for the lateral area on the wrist [1]. A triangular depression on the posterolateral aspect of the wrist joint is the 'Anatomical snuff box', which becomes more apparent on the thumb extension or ulnar deviation of the wrist joint. The base of this triangular area is at the wrist, and the apex is directed towards the thumb. Extensor tendons, while passing through this area, form the boundaries of this triangular area. Laterally, this area is bounded by two tendons. The lateral most tendon is the Abductor pollicis longus (APL), and the Extensor pollicis brevis (EPB) is medial to it. This area is bounded on the medial side by the Extensor pollicis longus tendon. The base of the first metacarpal, scaphoid, and trapezium bones forms the floor of this area. The radial artery acts as a content of this depression while passing obliquely deep to the extensor tendons. In coronary angiographic investigation or percutaneous intervention, an anatomical snuff box is approached to identify the distal part of the radial artery. [2] The roof bears the origin of the cephalic vein from the dorsal venous arch of the hand, accompanied by a superficial branch of the radial nerve. Noteworthy to mention about lateral most tendon, one of the 'outcropping muscles' i. e. Abductor pollicis longus arises from the posterior aspect of the ulna, adjoining interosseous membrane and the middle third of the dorsal aspect of the radius. It forms the tendon proximal to the wrist to be inserted into the radial side of the base of the first metacarpal bone [3]. The variation in the insertion of extensor tendons of the forearm in the hand and wrist is a fascinating phenomenon. Many studies in the literature report the variation in the insertion of these extensor tendons. A sound knowledge of such variations in extensor tendons is essential to understanding the consequence of tendon injury and further planning with practical implementation of repairs of the culprit. Variations are also crucial to a radiologist while reporting such cases and for an anatomist during dissecting. [4] Knowledge regarding the distal attachment of the abductor pollicis longus is crucial during grafting surgeries for the osteoarthritis of the base of the thumb. The duplicated or triplicated tendons of the muscle may be utilised as grafting material. [5] Therefore, it is of interest to investigate anatomical variations in APL tendon insertions in the Central Indian population. Hence, we measured the number of tendons of the muscle, distal attachment, and side to side variation.

## Material and Methods:

The present study was conducted at the Department of Anatomy, All India Institute of Medical Sciences, Nagpur, Maharashtra, India. 48 formaldehyde-fixed forearms of 24 Indian-origin cadavers between age groups ranging from 33 to 67 years donated to the department of Anatomy were included in this study. All upper limbs utilised for the study were externally normal. No signs of trauma, surgical incisions, or scars were identified on the surface. The muscles of the extensor compartment were dissected as per Cunningham's Manual of Practical Anatomy Volume 1: upper and lower limbs. The skin incision was given, followed by the dissection of superficial and deep fascia. The extensor retinaculum was identified. After identification, the extensor retinaculum was split, and the first compartment was carefully dissected. The tendon of Abductor Pollicis Longus was exposed and identified till the distal attachment. Variation in the insertion or the distal attachment of APL was noted if present.

## Results:

**Figure 1:**
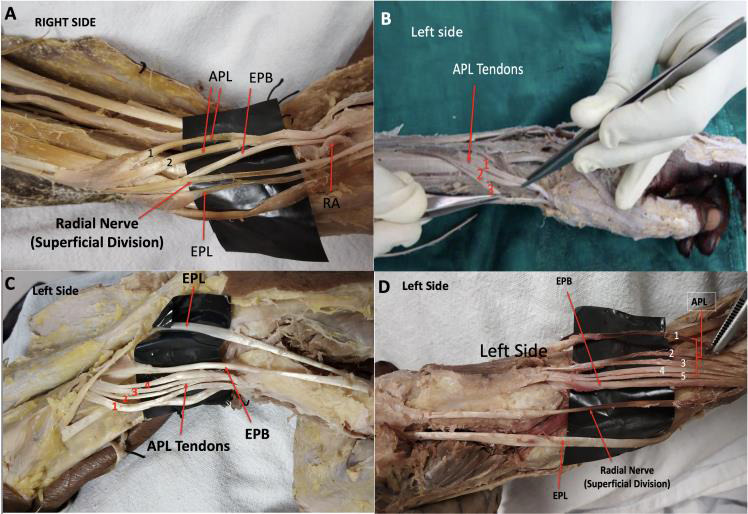
Variation in the tendons of the APL muscle is shown in the central Indian population samples. A - two tendons, B - three tendons, C -four tendons and D - 5 tendons. (EPB: Extensor pollicis brevis, EPL: Extensor pollicis longus)

APL muscle was found with the single tendon in 20 forearms, double in 9, triple in 8, and quadruple in 5 and the maximum number of tendons found was five in 6 forearms (Figure 1). The variation in the insertion of the APL tendon was also noted. In 93 % of hands (n=45), the APL tendon was inserted into the first metacarpal bone and in 7% (n=03), it was also inserted into the trapezium bone. No variation was noted in the Extensor pollicis brevis tendon, the companion tendon of APL.

Comparative measurements in relation to length, width, and thickness were done in limbs (Table 1). The mean length of APL tendon was found to be 68.1+ 15.9, whereas the mean length of AAPL was 68.9+19.2. No remarkable significance was identified in the length. The mean width of APL tendon was 5.4+4.2, whereas for AAPL, the width was 2.9+1.0. The mean width of AAPL was much lesser than the tendon of APL. The mean comparative thickness of APL was 2.6+0.7, whereas for AAPL was 1.5+0.6.

Comparative measurements of side-to-side differences in the tendon of APL and AAPL were identified in relation to length, width and thickness (Table 2). No significant differences were identified in side-to-side comparison of length and width. But side-to-side thickness in AAPL tendon was statistically significant compared to APL tendon.

## Discussion:

APL tendon-related anatomical abnormality may be asymptomatic or it manifest as a painful condition like de Quervain syndrome or tenosynovitis (DQT). Treatment options for severe occurrences of this illness include surgically decompressing the first extensor compartment or administering injectable therapy. Variations in the anatomy of the APL's tendons may be the cause of the DQT treatment's failure. [6] A report that described the wrists with de Quervain syndrome identified multiple APL tendons in 89% of the cadavers and 49% of the patients. The study showed that the multiple first extensor compartments may be predisposed to de Quervain syndrome. [7] de Quervain syndrome sometimes requires the tenosyno-vectomy procedure, where a close watch on accessory tendons, branching pattern of abductor pollicis longus and presence of atypical septum in first compartment becomes essential.

[8] Another study identified two APL tendons in 30 and three in 8 wrists. [9] A study on dimensions of multiple slips of APL tendons verified that the lateral tendon is the primary and the other medial tendons are accessory. [10] In another study, the APL tendon has four slips, and all are inserted into the fascia of the Abductor pollicis brevis muscle. This abnormal insertion was the reason for bilateral subluxation of the trapezio-metacarpal joint. [11] In one of the study, authors classified the distal attachment of the APL into three different subtypes like type I describes single distal attachment to base of first metacarpal, type II with bifurcated distal attachment and type III, main tensons attaching to base of first metacarpal, while accessory slips merges with other nearby tendons. [12] In another study, an independent muscle belly was found for accessory APL tendons with dual nerve supply, which was not in our data. Some authors reported ethnic variation in APL tendons' origin, insertion, and arrangement [5, 13]. In another study, authors noted no correlation between the number of tendinous slips, muscle bellies and innervation. [14] The tendons of the extensor carpi radialis longus and extensor carpi radialis brevis muscle are encased in a tunnel formed by the variations in the arrangement of APL muscle as reported elsewhere. [15] The length of the tendon of accessory APL required to be treated with arthroplasty for trapezio-metacarpal osteoarthritis is 6 cm. [5] Our data shows that the average length of multiple slips of APL tendons was 6.8 cm, suitable for graft material use. The incidence of trapezio-metacarpal arthritis was not shown to be influenced by the number of APL accessory slips. [16]

The precursor extensor muscle mass differentiates into a radial portion, subdivided into superficial and deep parts. The superficial portion differentiates and forms the Extensor Digitorum, Extensor carpi ulnaris and Extensor digiti minimi. The deep portion gives rise to Extensor pollicis longus, Abductor pollicis longus and Extensor pollicis brevis and Extensor indicis. Comparative anatomical studies on primates' results show that the deep portion undergoes marked variation. [17] The alteration in the deep portion of extensor muscle mass best explains the variation mentioned about the Abductor Pollicis Longus tendon in this study. In the early development period, the APL tendon has three strips of attachments. The dorsal strip is inserted into the first metacarpal bone, the middle into the trapezium and the palmer strip is attached to the Opponens pollicis. Afterwards, the palmer strip gets disconnected and receives a new connection with the Abductor pollicis brevis muscle. The persistence of this tendinous developmental pattern of APL may cause multiple tendons. [7] Molecular regulation of muscle development: Bone morphogenic protein 4 (BMP4) and fibroblast growth factors from lateral plate mesoderm, together with WNT proteins from adjacent ectoderm, signal VLL cells of the dermomyotome to express the muscle-specific gene MyoD. BMP4, secreted by ectoderm cells, induces the production of WNT proteins by the dorsal neural tube at the same time that low concentrations of sonic hedgehog proteins, secreted by the notochord and floor plate of the neural tube, reach the DML cells of the dermomyotome. Together, these proteins induce the expression of MYF5 and MyoD in these cells. Both MyoD and MYF5 are members of a family of transcription factors called myogenic regulator factors (MRFs), and this group of genes activates pathways for muscle development [18]. During the fourth week of development (on the 26th day), a limb bud derived from mesodermal tissue from somites and the lateral plate mesoderm begins to form the upper limb. The formation of the upper limb is in the proximo-distal direction. The formation of the upper limb is controlled by many signalling centres like the Apical ectodermal ridge (AER), Zone of polarizing activity (ZPA), and Wnt pathway. These signalling centres produce specific factors that are going to control differentiation. AER controls proximo-distal signalling by inducing differentiation of underlying mesoderm and apoptosis of interdigital tissue. ZPA produces sonic hedgehog protein (SHH), which controls radioulnar limb formation. The Wnt pathway regulates the ventral and dorsal limb axis. Disruption of any of the processes, as mentioned above, can lead to anomalies in the upper limb [19]. Reports showed APL tendon inserting on the thenar aponeurosis is a good donor for thumb opposition repair. [20] The functional nature of each tendon or tendinous slip is still unknown, but the ontogenesis of the APL muscle may explain these numerical and insertional differences as well as the thumb's seemingly ever-evolving muscular apparatus [21].

## Conclusion:

Data shows that there are accessory slips in the APL tendon. This variation differs from the known standard description. Thus, this data provide awareness on such variation amongst the operating surgeons for consideration during treatment in the management of the diseased and other linked treatment procedures.

## Financial support & sponsorship:

Nil

## Figures and Tables

**Table 1:** Comparative length, width & thickness measurements of APL, AAPL (in mm)

	**Comparative Length**			**Comparative width**			**Comparative thickness**		
	Mean ±SD	Minimum	Maximum	Mean ±SD	Minimum	Maximum	Mean ±SD	Minimum	Maximum
APL	68.1 ± 15.9	30	126	5.4 ± 4.2	1.9	9.7	2.6 ± 0.7	1	3.6
AAPL	68.9 ± 19.2	5.6	123	2.9 ± 1.0	1.2	6.1	1.5 ± 0.6	0.6	2.4
APL= abductor pollicis longus tendon; AAPL=accessory abductor pollicis longus tendon

**Table 2:** Comparative measurement of side-to-side differences in length, width, and thickness of APL and AAPL (in mm)

		**Side to side differences in length**		**Side to side differences in width**		**Side to side differences in thickness**	
APL	Right	68.2 ± 16.12	P value=0.955	5.7 ± 3.8	P value= 0.807	2.3 ± 0.9	P value= 0.23
	Left	67.9 ± 15.93		5.2 ± 4.0		2.5 ± 0.7	
AAPL	Right	68.27 ± 19.6	P value= 0.941	2.7 ± 0.9	P value= 0.175	1.6 ± 0.4	P value= 0.001*
	Left	68.63 ±19.3		2.8 ± 1.2		1.3 ± 0.8	
APL= abductor pollicis longus tendon
AAPL=accessory abductor pollicis longus tendon
*P<0.05 is statistically significant
